# Consortium of Probiotics Attenuates Colonization of *Clostridioides difficile*

**DOI:** 10.3389/fmicb.2019.02871

**Published:** 2019-12-12

**Authors:** Xianping Li, Qiongfang Chu, Yuanming Huang, Yuchun Xiao, Liqiong Song, Siyi Zhu, Ying Kang, Shan Lu, Jianguo Xu, Zhihong Ren

**Affiliations:** ^1^State Key Laboratory for Infectious Disease Prevention and Control, National Institute for Communicable Disease Control and Prevention, Collaborative Innovation Center for Diagnosis and Treatment of Infectious Diseases, Chinese Center for Disease Control and Prevention, Changping, China; ^2^Research Units of Discovery of Unknown Bacteria and Function (2018 RU010), Chinese Academy of Medical Sciences, Beijing, China; ^3^Beijing Dongcheng District Longtan Community Health Center, Beijing, China

**Keywords:** *C. difficile*, probiotics, *Lactobacillus*, *Bifidobacterium*, bile acids, short-chain fatty acids

## Abstract

*Clostridioides difficile* infection (CDI) is increasing morbidity and mortality rates globally. Fecal microbiota transplantation (FMT), an effective therapy for eliminating *Clostridioides difficile* (*C. difficile*), cannot be used extensive due to a range of challenges. Probiotics thus constitutes a promising alternative therapy. In our study, we evaluated the effect of consortium of probiotics including five *Lactobacilli* strains and two *Bifidobacterium* strains on the colonization of toxigenic BI/NAP1/027 *C. difficile* in a mouse model. The results of 16S rRNA sequencing and targeted metabolomics showed the consortium of probiotics effectively decreased the colonization of *C. difficile*, changed the α- and β-diversity of the gut microbiota, decreased the primary bile acids, and increased the secondary bile acids. Spearman’s correlation showed that some of the OTUs such as *Akkermansia*, *Bacteroides*, *Blautia et al*. were positively correlated with *C. difficile* numbers and the primary bile acids, and negatively correlated with the secondary bile acids. However, some of the OTUs, such as *Butyricicoccus*, *Ruminococcus*, and Rikenellaceae, were negatively correlated with *C. difficile* copies and the primary bile acids, and positively correlated with the secondary bile acids. In summary, the consortium of probiotics effectively decreases the colonization of *C. difficile*, probably via alteration of gut microbiota and bile acids. Our probiotics mixture thus offers a promising FMT alternative.

## Introduction

*C. difficile* is the major pathogenic bacterium causing antibiotic-associated diarrhea (CDAD). Additionally, *C. difficile* infection (CDI), one of the most common nosocomial infections, has demonstrated sustained growth in recent years (Louh et al., 2017). CDI has increased considerably in magnitude and poses serious threats to both health and economy. For example, 453,000 new cases are reported each year in the United States, with the cost of care reaching $4.8 billion (Dieterle and Young, 2017). Antibiotic treatment is still the first choice for CDI. However, higher recurrence rates have forced researchers to seek alternative therapeutic methods. FMT, is a powerful and effective therapy to eliminate *C. difficile* colonization and restore the composition of the gut microbiota both in a mouse model of recurrent *C. difficile* infection (rCDI) (Seekatz et al., 2015) and in patients with rCDI (Borody and Khoruts, 2011; van Nood et al., 2013). However, the application of FMT has certain limitations. Firstly, difficulties exist in the recruitment of healthy donors (Rohlke and Stollman, 2012) and the standardization of donated stool testing processes. Secondly, some pathogens and opportunistic pathogens are not detectable due to the limitations of the testing technology. Two cases of patients contracting norovirus following FMT have been reported (Schwartz et al., 2013). FMT is also correlated with the development of peripheral neuropathy, rheumatoid arthritis, and idiopathic thrombocytopenic purpura (Brandt et al., 2012). In view of the limitation, FMT is not administered as initial treatment for CDI (Mullish et al., 2018). However, FMT is still recommended in the treatment of rCDI or refractory CDI due to irreplaceable efficacy (Mullish et al., 2018; Allegretti et al., 2019). Therefore, a probiotics mixture with clear ingredient would be a promising bacteria therapy of CDI or rCDI. Probiotics, defined as “live microorganisms that, when administered in adequate amounts, confer a health benefit on the host” (FAO and WHO, 2001), have been proposed to restore the modifications of gut microbiota caused by antibiotics or infections (Reid et al., 2011). Bacteriotherapy, as a promising and effective therapy for CDI, has been widely studied in *C. difficile*-infected mouse models and patients with CDI (Lawley et al., 2012; Petrof et al., 2013; Buffie et al., 2015; De Wolfe et al., 2018). Petrof et al. constructed a stool substitute constituted by 33 different purified intestinal bacteria for the treatment rCDI, which showed the synthetic mixture may be an effective method to replace FMT especially for rCDI (Petrof et al., 2013). A meta-analysis of 8,672 cases uncovered moderate beneficial evidence for prevention of CDAD by probiotics (Goldenberg et al., 2017; Suez et al., 2019), another meta-analysis concluded that only *S. boulardii* was effective against *C. difficile* (McFarland, 2006).

Many studies have also demonstrated that *Lactobacillus* and *Bifidobacterium* are associated with colonization resistance against *C. difficile* (Lawley et al., 2012; Petrof et al., 2013; Valdes-Varela et al., 2016; Martz et al., 2017; De Wolfe et al., 2018; Vedantam et al., 2018). *Bifidobacterium longum* JDM301, a widely used commercial probiotic strain, can inhibit *C. difficile* growth and degrade TcdA and TcdB, and the author further proved that the exertion of inhibition of *B. longum* is dependent on an acidic pH (Wei et al., 2018). We speculate that *Lactobacilli* with acid- and bile salt-tolerance which ensured to reach the intestine, could provide an acidic microenvironment in which *Bifidobacteria* can maximize the suppression of *C. difficile*. Moreover, various lactobacilli may produce various SCFAs, which benefit for intestinal epithelial cell. Lema et al. (2001) studied the efficacy of monostrain and multispecies probiotics and indicated that a multispecies probiotic demonstrated the best clearance effect of *E. coli* O157:H7 from lambs. VSL#3, a mixture of bacteria consisting of four strains of *Lactobacillus*, and three strains of *Bifidobacterium*, and one strain of *Streptococcus salivarius* subsp. *Thermophiles*, was demonstrated to prevent antibiotic associated diarrhea (AAD) and CDAD (Selinger et al., 2013). Therefore, we selected a consortium of probiotics, including five *Lactobacilli* strains (*Lactobacillus gasseri* Y20, *Lactobacillus murinus* Y74, *Lactobacillus plantarum* HT47, *Lactobacillus reuteri* HT119, and *Lactobacillus plantarum* HT121) derived from different hosts and two *Bifidobacterium* standard strains [*Bifidobacterium adolescentis* (ATCC15703) and *Bifidobacterium infantis* (ATCC 15697)], as a candidate to verify the anti-bacterium effect in a *C. difficile*-infected mouse model.

The precise mechanism of FMT or probiotics in decreasing *C. difficile* remains to be fully elucidated. Some studies have indicated that the restoration of gut microbiota diversity is a major mechanism of the prevention of CDI (Seekatz et al., 2015; Khanna et al., 2016; Kellingray et al., 2018), while other studies have showed that the restoration of bile acids plays a pivotal role in decreasing *C. difficile* (Weingarden et al., 2014; Buffie et al., 2015; Yoon et al., 2017). The purpose of our study was to determine whether the consortium of our probiotics could protect against hyper virulent ribotype 027 *C. difficile* infection in a mouse model. Additionally, we aimed to identify the potential mechanisms associated with the decrease in *C. difficile* based on the analysis of the gut microbiota composition and bile acid profile.

## Materials and Methods

### Ethics Statement

The study was approved by the Ethics Review Committee of the National Institute for Communicable Disease Control and Prevention at the Chinese Center for Disease Control and Prevention (Beijing, China).

### Screening and Cultivation of Candidates

A total of five *Lactobacilli* strains, isolated from the feces of healthy breast-feeding infants and Tibet wild marmots, were screened and administrated in our study. *Lactobacilli* with acid- and bile salt-tolerance were isolated and screened. In brief, 100 μL of feces suspension, diluted by serial 10-fold dilution, was evenly coated on a Man-Rogosa-Sharpe (MRS) plate and cultured in a 37°C carbon dioxide incubator (Thermo Scientific Forma CO_2_, United States) for 2 days. *Lactobacilli* were identified using biochemical methods and 16S rRNA sequencing. The acid and bile salt-tolerance assays were performed on an MRS plate containing 0.3% bovine bile salt at pH = 2. According to the results of the salt and bile tolerance assays, five candidate strains were selected and named *Lactobacillus gasseri* Y20, *Lactobacillus murinus* Y74, *Lactobacillus plantarum* HT47, *Lactobacillus reuteri* HT119, and *Lactobacillus plantarum* HT121, the first two from infants and the others from marmots. *Bifidobacterium adolescentis* (ATCC15703) and *B. infantis* (ATCC 15697), purchased from the American Type Culture Collection (ATCC, United States), were routinely grown in MRS medium in an anaerobic chamber (Electrotek AW200SG, United Kingdom). Each strain of seven probiotics was washed with sterile phosphate-buffered saline (PBS) and resuspended in sterile PBS at 3 × 10^8^ CFU/mL, following which they were mixed into equal parts to form a consortium of probiotics (approximate total content of 2 × 10^9^ CFU/mL) for further evaluation of their efficacy in inhibiting *C. difficile* colonization in a mouse model.

### CDI Mouse Model and Intervention

Female C57BL/6J wild-type mice (7–8 weeks old) ranging from 18 to 20 g were purchased from Beijing Vital River Laboratory Animal Technology Co., Ltd (Beijing, China). A total of 32 mice, 8 in control group, 12 in PBS or probiotics, were used in our experiments. And all mice were conducted in accordance with the protocols approved by the Welfare and Ethical Inspection in Animal Experimentation Committee at the Chinese CDC. All mice were housed in groups of 4 per cage with autoclave bedding, food, and water in the China CDC Animal Center. The mice were permitted free access to water and food under a 12 h light cycle. After a 7 days adaptation period, the mice were randomly divided into three groups: control mice without any treatment, PBS-treated mice infected with *C. difficile* (PBS group), and probiotics-treated mice infected with *C. difficile* (probiotics group).

To infect the animals, an antibiotic mixture of kanamycin (0.4 mg/mL), gentamicin (0.035 mg/mL), colistin (850 U/mL), metronidazole (0.215 mg/mL), and vancomycin (0.045 mg/mL) was added to the drinking water for 5 consecutive days (Chen et al., 2008). The antibiotic mixture was then stopped and replaced with autoclave water. Two days after antibiotics cessation, the mice received intraperitoneal injections of clindamycin (20 mg/kg), following which they were challenged 1 day later with 10^7^ CFU of toxigenic BI/NAP1/027 *C. difficile* strain (ATCC^®^ BAA-1870^TM^) (kindly supplied by Dr. Wu Yuan of the Chinese CDC). One day after *C. difficile* infection, 200 μL PBS alone or 200 μL PBS containing 4 × 10^8^CFU consortium of probiotics was administrated to the *C. difficile*-infected mice by oral gavage for 9 consecutive days.

### Histopathological Analysis

Colonic tissue was collected and fixed in 10% formalin solution. Two days later, the tissue was embedded in paraffin and then sliced into 4 μm thick sections, which were stained with hematoxylin and eosin. A histological graded scoring system was used to assess pathological tissue inflammation as follows: 0, normal; 1, inflammatory cells increase in lamina propria; 2, inflammatory cells increase in submucosa; 3, plenty of inflammatory cell mass. Stained sections were examined and evaluated blindly by a certified pathologist (Chen et al., 2008; Wei et al., 2018).

### Fecal DNA Extraction and *C. difficile*, *Lactobacillus* spp., and *Bifidobacterium* spp. Detection by Real-Time Quantitative PCR (RTq-PCR)

The feces were collected on days 1, 6, and 10 after *C. difficile* infection. DNA was extracted using the QIAamp DNA Stool Mini Kit (Qiagen, Germany) according to manufacturer’s instructions. The oligonucleotide primers and probe were synthesized by Sangon Biotech Co., Ltd. (Shanghai, China). The 20 μL reactions contained 10 μL of TaqMan mix (Takara, China), 200 nM of probe, 250 nM of each primer, 0.4 μL of DyeII, and 1.5 μL of DNA from a fecal sample. Amplifications were performed in an ABI 7500 Real-Time PCR System (ABI, United States) under the following conditions: 1 cycle at 94°C for 30 s, followed by 40 cycles of denaturation at 95°C for 10 s and annealing at 55°C for 30 s. For *Bifidobacterium* spp., the annealing temperature was 62°C. The number of *C. difficile, Lactobacillus* spp., and *Bifidobacterium* spp. copies were calculated based on their standard curves, respectively. The primers and probes were listed on Supplementary Table S1 (Belanger et al., 2003; Penders et al., 2005; Haarman and Knol, 2006).

### 16S rRNA Sequencing and Data Analysis

Fresh feces, collected promptly from the mice on day 9 after the intervention, were frozen immediately and stored at −70°C. Fecal DNA was extracted using the QIAamp DNA Stool Mini Kit (Qiagen, Germany) following the manufacturer’s instructions. DNA concentration and integrity were measured, and the qualified samples were used to construct a database, following which they were sequenced and analyzed by Beijing Genomics Institute Co., Ltd. (Beijing, China). Briefly, the extracted DNA was used as a template to amplify the V4 hypervariable region of 16S rRNA genes using the modified universal bacterial primer pairs 515F (GTGCCAGCMGCCGCGGTAA) and 806R (GGACTACHVGGGTWTCTAAT). Pyrosequencing of the PCR amplicons and quality control of the raw data were performed on the Illumina MiSeq PE250 platform. The raw sequences were subjected to USEARCH v10.0.240 (Edgar, 2013) and Vsearch v2.8.1 (Rognes et al., 2016) for quality assurance and OTU picking. Briefly, the raw sequences were first demultiplexed, then the following reads merged into paired reads, and the primer was stripped. For quality filtering, merged reads with the specified number of expected errors per base threshold larger than 0.01 or read length shorter than 160 was discarded. The quality filtered reads were dereplicated into unique sequences and the chimeras were filtered out from OTUs by UCHIME (v4.2.40) (Edgar et al., 2011). OTU representative sequences were taxonomically classified using Ribosomal Database Project (RDP) Classifier v.2.2 trained on the Greengenes database (V201305) and RDP database (Release 11_5, 20160930), using 0.80 confidence values as cutoff. α-diversity, including the richness index, Shannon_e index, and Chao1 index, and principal coordinates analysis (PCoA), were calculated and visualized in R software (v3.1.1). Linear discriminant analysis (LDA) effect size (LEfSe) analysis^1^ was used to identify key differential OTUs between the groups based on nonparametric factorial Kruskal–Wallis and Wilcoxon rank sum tests at a significance level of 0.05. The threshold on the logarithmic LDA score for discriminative features was set at 2.0 (Segata et al., 2011). The original data have been submitted to SRA, and can be downloaded from the NCBI SRA database (accession number: SRP200838).

### Quantification Analysis of Bile Acids by Liquid Chromatography-Mass Spectrometry (LC-MS)

Feces, collected on day 9 of the intervention, were stored at −70°C, and the bile acids were detected by the Beijing Bio-Tech Pack Technology Company Ltd. (Beijing, China). Briefly, feces (100–200 mg) were suspended in 1 mL methanol and extracted by vortexing for 60 min, following centrifugation at 13,200 r/min for 10 min. A total of 500 μL of supernatant was centrifuged and volatilized by freeze-drying under vacuum, followed by the addition of 150 μL methanol to the Eppendorf tube to dissolve the sediment. The supernatant was detected on a liquid chromatograph (LC, UltiMate 3000 UHPLC, Thermo Scientific^TM^, United States) and mass spectrometer (MS, Q Exactive^TM^, Thermo Scientific^TM^, United States). The concentration of the bile acids was calculated according to the peak areas of the bile acids and external standards.

### Quantification Analysis of SCFAs by Gas Chromatography-Mass Spectrometry (GC-MS)

Feces, collected on day 9 of the intervention, were stored at −70°C, and the SCFAs were detected by the Beijing Bio-Tech Pack Technology Company Ltd. Briefly, feces (200 mg) were suspended in 500 μL water (pH = 1), and 500 μL ethyl acetate was added into the feces suspension, following which 1 mL was mixed by vortexing for 30 min and then centrifuged at 1,200 r/min for 10 min. The supernatant was subjected to GC-MS (Agilent Technologies, Santa Clara, CA, United States) (Gao et al., 2010). The operating parameters were as follows: initial temperature of 50°C for 1 min, followed by a 12°C/min rate increase to 170°C and then a 20°C/min increase to 230°C, at which it was maintained for 3 min. The inlet temperature was 250°C, the split ratio was 1/50, the flow rate was 1.2 mL/min, and the detector temperature was 230°C. The carrier gas was He.

### mRNA Extraction and RTq-PCR

Total RNA of the colonic tissue was extracted using TRIzol (Invitrogen, United States) and reverse-transcribed to generate cDNA by reverse transcriptase (Promega, United States) according to the manufacturer’s instructions. The cDNA products were subjected to RTq-PCR in an ABI 7500 Real-Time PCR System (ABI, United States). The 20 μL reactions contained 10 μL of SYBR Green PCR Master Mix (Takara, China), 300 nM of each primer, 0.4 μL of DyeII, and 2 μL of cDNA product. The amplification reactions were performed under the following conditions: 1 cycle at 95°C for 30 s, 60°C for 15 s, and then 95°C for 15 s, followed by 40 cycles of 95°C for 10 s and 60°C for 32 s. Dissolution curve analysis was performed after each cycle at 95°C for 15 s and 60°C for 60 s to avoid the interference of primer dimers and secondary structure. The folds were calculated using the comparative 2^–ΔΔCt^ method, β-actin was used as an internal control. The primers are listed in Supplementary Table S1.

### Statistical Analysis

Statistical analysis was performed with GraphPad Prism (v.5.0) and R (v. 3.2.5). Comparisons of the number of copies of *C. difficile* in the feces and the level of SCFAs between the two groups were analyzed using unpaired *t*-tests, while the histological scores data were analyzed using nonparametric tests. The α-diversity, the relative abundance of the OTUs, the level of bile acids, the copies of *Lactobacillus* spp. and *Bifidobacterium* spp., and the level of mRNA expression in the colon were analyzed using one-way analysis of variance (ANOVA). The data of PCoA were analyzed using nonparametric MANOVA based on Adonis. The correlations of the number of copies of *C. difficile* in the feces, the relative abundance of the OTUs, the level of bile acids, and the concentrations of the SCFAs were analyzed by Spearman’s correlation tests. *P* < 0.05 was considered statistically significant.

## Results

### A Consortium of Probiotics Effectively Attenuated *C. difficile* in the Feces and Decreased the mRNA Expressions of IL-12 and Reg3-β in Colonic Tissue in *C. difficile*-Infected Mouse Model

**FIGURE 1:**
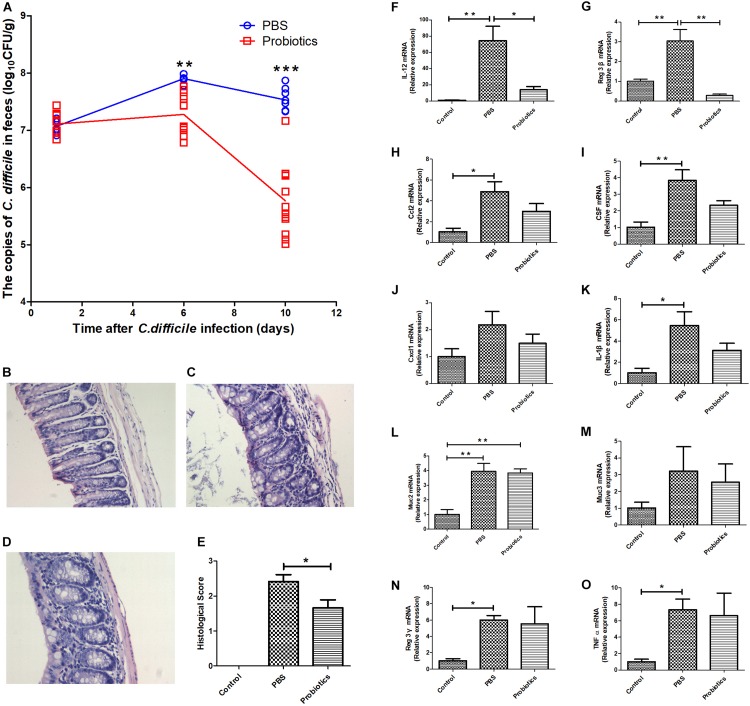
A consortium of probiotics effectively attenuated *C. difficile* in the feces and decreased the mRNA expressions of IL-12 and Reg3-β in colonic tissue in *C. difficile*-infected mouse model. **(A)** Mouse feces were collected at 1, 6, and 10 days after *C. difficile* infection. The copies of *C. difficile* in the feces were measured by real-time quantitative PCR. The copies of *C. difficile* were significantly decreased at day 6 (*P*< 0.01), and by day 10, the gap had widened further (*P*< 0.001). The data were analyzed using an unpaired *t*-tests, with *n* = 6 for each group. **(B–D)** Representative images of 12 Hematoxylin-Eosin staining sections, respectively, representing the control, PBS, and probiotics groups. **(B)** Colonic tissue of the untreated mice, and no inflammatory cell infiltration was observed. In contrast, there was great lymphocyte infiltration in the lamina propria and submucosa of the colonic tissue in **(C).** Additionally, the number of lymphocytes was lower in **(D)** than in **(C)**, and the lymphocyte infiltration in **(D)** was mainly in the lamina propria. **(E)** Histological scores of the colon. The probiotics group was significantly lower in histological scores based on lymphocyte infiltration compared with the PBS group. **(F–O)** Mice were euthanized at 10 days post-treatment. The mRNA levels of the related inflammatory cytokines and intestinal mucous proteins were measured by RTq-PCR in the colon. **(F–O)** Represent IL-12, Reg3-β, Ccl2, CSF, Cxcl1, IL-1β, Muc2, Muc3, Reg3-γ, and TNFα, respectively. The data of E was analyzed using nonparametric tests (*P*< 0.05). Statistical analysis **(F–O)** were performed using one-way ANOVA (*P* < 0.05). ^∗^*P*< 0.05, ^∗∗^*P*< 0.01, ^∗∗∗^*P*< 0.001.

As previously described, C57/BL6/J mice were administrated with an antibiotic cocktail and orally infected with 10^6^ *C. difficile* BI/NAP1/027. The mouse feces were collected at day 1 post-infection. There was no significant difference in the copies of *C. difficile* in the feces between the PBS and probiotics groups based on RTq-PCR (*P*> 0.05, 7.07 ± 0.14 vs. 7.17 ± 0.17), which indicated that the models of the *C. difficile* infected-mice were established successfully. The copies of *C. difficile* in the feces of the probiotics group were significantly lower at 6 days post-infection (*P* < 0.01, 7.90 ± 0.06 vs. 7.28 ± 0.34) and further lower at 10 days post-infection compared to those in PBS group (*P* < 0.001, 7.53± 0.20 vs. 5.77± 0.62) (Figure 1A). The mice treated with probiotics also exhibited less colonic inflammation and lymphocyte infiltration compared with the PBS treated-mice (Figures 1C–E). Infection with *C. difficile* resulted in a fair amount of lymphocytic infiltration in the lamina propria and submucosa of the colonic tissue (Figure 1C), and the 7-probiotic consortium treatment reduced the number of infiltrated lymphocytes (Figure 1D). No lymphocyte infiltration was observed in the untreated colonic tissue of the mice (Figure 1B).

To investigate the role of inflammatory response in the intestinal tissue in decreasing *C. difficile* numbers, we performed RTq-PCR to measure the mRNA expression of related inflammatory cytokines, i.e., IL-1β, IL-12, Ccl2, CSF, Cxcl1, Reg3-β, Reg3-γ, and TNFα and intestinal mucous proteins Muc2 and Muc3 at day 10 post-infection. The mRNA levels of IL-12 and Reg3-β in probiotic-treated mice were significantly lower than PBS-treated mice (Figures 1F,G). However, the other genes did not differ significantly between the two groups (Figures 1H–O).

### The α- and β-Diversity of the Gut Microbiota Were Different Among Three Groups

**FIGURE 2:**
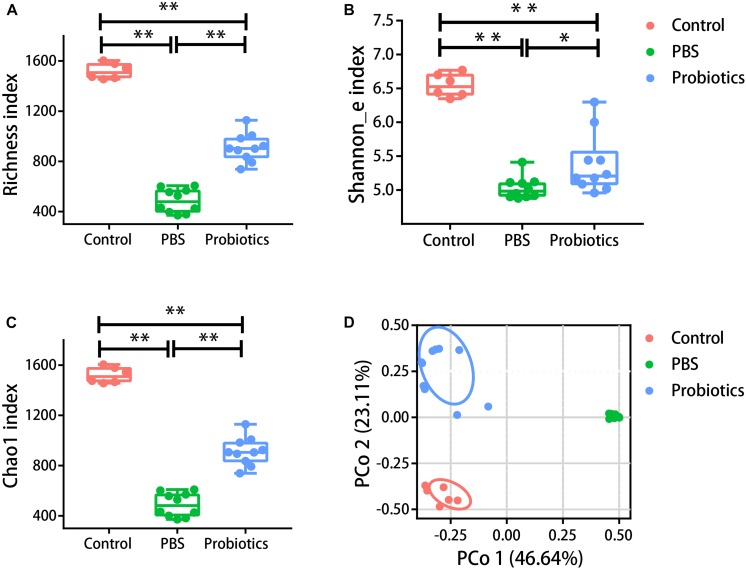
The α- and β-diversity of the gut microbiota were different among three groups. Mouse feces were collected and DNA was extracted for 16S rRNA gene sequencing 10 days post-infection. Analysis of the 16S rRNA gene sequences (variable regions 4) derived from the fecal pellets of the control group (*n* = 6), PBS group (*n* = 10), and probiotics group (*n* = 10). **(A–C)** Represent the Richness index, Shannon_e index, and Chao1 index, respectively. Probiotics treatment significantly increased the levels of the Richness index, Shannon_e index, and Chao1 index compared to the PBS treatment. The data were analyzed using one-way ANOVA (*P* < 0.05). **(D)** Is the PCoA plot of the intestinal microbiota based on unweighted UniFrac metrics. The data were analyzed using nonparametric MANOVA based on Adonis (*P* < 0.05). PCO 1 accounted for 46.64% of the variation, and there were no significant differences between the control and probiotics groups in PCO 1. The data were analyzed using Kruskal-Wallis test (*P* < 0.05). ^∗^*P*< 0.05, ^∗∗^*P*< 0.01.

α-diversity in our study was assessed using three metrics: the Richness index (the count of unique OTUs), the Shannon_e index, and the Chao1 index (estimating the community richness). As expected, the values of the Richness index (Figure 2A), Shannon_e index (Figure 2B), and Chao1 (Figure 2C) index were significantly lower in the mice of PBS group compared to the untreated mice (*P* < 0.05). The values of the three metrics of the probiotics group increased significantly in probiotics group compared with those of the PBS group (*P* < 0.05). To examine the β-diversity, PCoA was performed on the basis of the UniFrac distances. The PCoA showed that the intestinal microbiota structure showed three differential groups (*P* < 0.05). The differences related to probiotics treatment were mainly observed along the first principal coordinate, which accounted for the largest proportion of the variation at 46.64% (Figure 2D). There was no markedly difference along the first principal coordinate between the control mice and probiotics group (*P* > 0.05).

### The Relative Abundances of Firmicutes and Bacteroidetes Were Higher and Those of Proteobacteria and Verrucomicrobia Were Lower in Control or Probiotics Compared to PBS Group

To identify the OTUs that were involved in the differences in the probiotics group, we conducted LEfSe analysis. The relative abundances of the significantly differential OTUs are indicated in Supplementary Figure S1A. As indicated in Supplementary Figures S1B,C, there was no significant difference in the relative abundances of Firmicutes, Bacteroidetes, Proteobacteria, and Verrucomicrobia between control and probiotics group. However, PBS-treated mice had significantly lower relative abundances of Firmicutes and Bacteroidetes and higher relative abundances of Proteobacteria and Verrucomicrobia compared to control or probiotics-treated mice (Supplementary Figures S1B–E).

### Correlation Between the Key Differential OTUs and *C. difficile* Numbers

**FIGURE 3:**
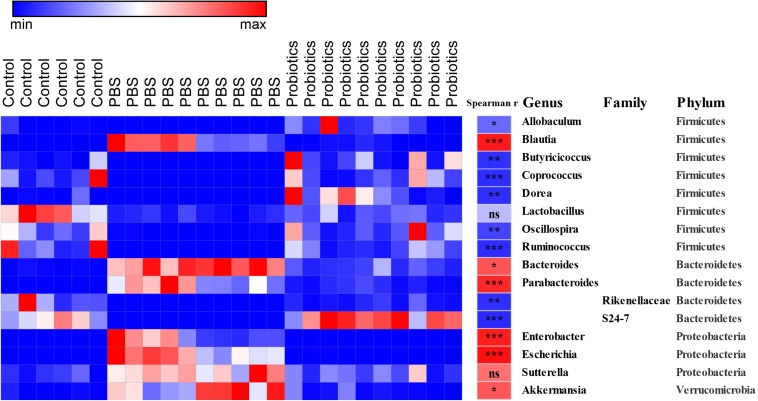
Heat map of the discriminant key OTUs in response to probiotic treatment determined by LEfSe analysis with a threshold *P*-value of 0.05 and Spearman’s correlation between the differential OTUs and the number of *C. difficile*. Sixteen identified OTUs are displayed in the heat map. Spearman’s r of the abundance of the discriminant OTUs and the *C. difficile* number was conducted and is indicated in the middle column. Taxonomic assignments at the genus, family, and phylum levels are shown in the right columns, respectively. Data were analyzed using Spearman’s correlation analysis. The *r*-values are denoted using graduated colors, and red and blue grids indicate positive and negative correlations, respectively, ^∗^*P* < 0.05, ^∗∗^*P* < 0.01, ^∗∗∗^*P* < 0.001.

Sixteen bacterial OTUs differed in relative abundance among the 3 groups of mice (Figure 3). Seven OTUs were higher in the *C. difficile*-infected mice, including *Blautia*, *Bacteroides*, *Parabacteroides*, *Enterobacter*, *Escherichia*, *Sutterella*, and *Akkermansia*, while 9 OTUs were lower, including *Allobaculum*, *Butyricicoccus*, *Coprococcus*, *Dorea*, *Lactobacillus*, *Oscillospira*, and *Ruminococcus*, and the families Rikenellaceae and S24-7.

To demonstrate whether the key differential OTUs of the intestinal microbiota were associated with the numbers of *C. difficile* in the feces, we performed an association analysis using different OTUs obtained from the LEfSe analysis based on the Spearman’s rank correlation coefficient (Figure 3). At the family level, Rikenellaceae (*r* = −0.82, *P* < 0.01) and S24-7 (*r* = −0.83, *P* < 0.001) showed a strong negative correlation with the *C. difficile* number in the feces. At the genus level, *Allobaculum* (*r* = −0.59, *P* < 0.05), *Butyricicoccus* (*r* = −0.81, *P* < 0.01), *Coprococcus* (*r* = −0.82, *P* < 0.001), *Dorea* (*r* = −0.82, *P* < 0.01), *Oscillospira* (*r* = −0.73, *P* < 0.01), and *Ruminococcus* (*r* = −0.82, *P* < 0.001) were significantly negatively correlated with *C. difficile* number. At the genus level, *Blautia* (*r* = 0.90, *P* < 0.001), *Bacteroides* (*r* = 0.67, *P* < 0.01), *Parabacteroides* (*r* = 0.84, *P* < 0.001) *Enterobacter* (*r* = 0.88, *P* < 0.001), *Escherichia* (*r* = 0.94, *P* < 0.001), and *Akkermansia* (*r* = 0.66, *P* < 0.01) had significantly and strongly positively correlated with *C. difficile* number. *Lactobacillus* (*r* = −0.27, *P* > 0.05) and *Sutterella* (*r* = 0.56, *P*> 0.05) exhibited a weak negative/positive correlation without statistical significance. The negative correlations were mainly observed in genera belonging to Firmicutes, while the positive correlations were mainly observed in Proteobacteria and Verrucomicrobia.

### The Primary Bile Acids Were Lower, and Secondary Bile Acids Were Higher in Feces of Probiotics or Control Group Than Those of PBS Group

**FIGURE 4:**
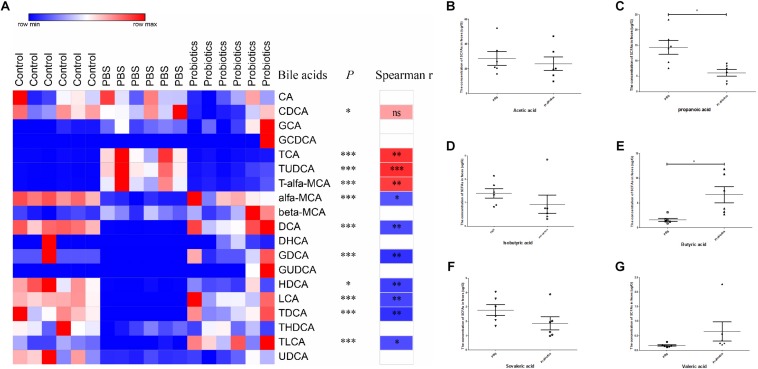
Heat map of the bile acids and comparison of the concentration of SCFAs. **(A)** Heat map and correlation analysis of the bile acids based on Spearman’s correlations between the level of bile acids and the *C. difficile* numbers. Mouse feces were collected 10 days post-infection. Bile acids in the feces were measured by LC-MS. Nineteen bile acids are displayed in the heat map in the left columns, with the name of the bile acids and the *P*-values indicated in the middle columns. Spearman’s rank correlation coefficients are indicated on the right of the columns. The *r*-values are denoted by graduated colors, and the red and blue grids indicate positive and negative correlations, respectively. **(B–G)** Represents a comparison of the concentration of SCFAs in the feces between the PBS and probiotics group. Mouse feces were collected at 10 days post-infection. SCFAs in the feces were measured using GC-MS. Six SCFAs were measured and compared between the PBS and probiotics group. The data were analyzed using one-way ANOVA or unpaired *t*-tests (*P* < 0.05). All data are expressed as mean ± SD (*n* = 6). ^∗^*P* < 0.05, ^∗∗^*P* < 0.01, ^∗∗∗^*P* < 0.001.

To measure the bile acid concentrations of the gut microbiota, mouse feces were collected at 10 days post-infection. A total of 19 bile acids were measured, and no significant differences in CA, GCA, GCDCA, beta-MCA, DHCH, GUDCA, THDCA, and UDCA were detected among three groups. The levels of CDCA, TCA, TUDCA, and T-α-MCA were significantly lower in the probiotics or control group compared to the PBS group (^∗^*P* < 0.05, ^∗∗∗^*P* < 0.001) (Figure 4A and Supplementary Table S2) and all these bile acids were primary bile acids (TUDCA is a secondary bile acid in humans but is a primary bile acid in mice). The level of α-MCA, DCA, GDCA, HDCA, LCA, TDCA, and TLCA were significantly higher increased in the probiotics or control group compared to the PBS group (^∗^*P* < 0.01, or ^∗∗∗^*P* < 0.001) (Figure 4A and Supplementary Table S2)and six of seven higher level of bile acids were secondary bile acids beside α-MCA.

To demonstrate whether the discrepant bile acids were associated with *C. difficile* number in the feces, we performed an association analysis based on the Spearman’s rank correlation coefficient. The level of three primary bile acid TCA (*r* = 0.80, *P*< 0.01), TUDCA (*r* = 0.83, *P* < 0.001), and T-α-MCA (*r* = 0.76, *P* < 0.01) indicated a significant positive correlation with the number of *C. difficile* in the feces. The concentration of most secondary bile acids including DCA (*r* = −0.71, *P* < 0.01), GDCA (*r* = −0.81, *P* < 0.01), HDCA (*r* = −0.76, *P* < 0.01), LCA (*r* = −0.73, *P*< 0.01), TDCA (*r* = −0.81, *P* < 0.01), and TLCA (*r* = −0.70, *P*< 0.05) and only one primary bile acid α-MCA (*r* = −0.67, *P* < 0.05) were significantly negatively correlated with the *C. difficile* numbers in the feces.

To measure the level of SCFAs of the intestinal microbiota, mouse feces were collected at day 10 post-infection and six SCFAs were measured. The levels of propanoic acid and butyric acid, which respectively decreased and increased significantly (*P* < 0.05) in the probiotics group compared to PBS group, the level of acetic acid, propanoic acid, isobutyric acid, butyric acid, isovaleric acid, and valeric acid did not differ significantly between the two groups (Figures 4B–G).

### Correlation Analysis of the Key Differential OTUs and Bile Acids

**FIGURE 5:**
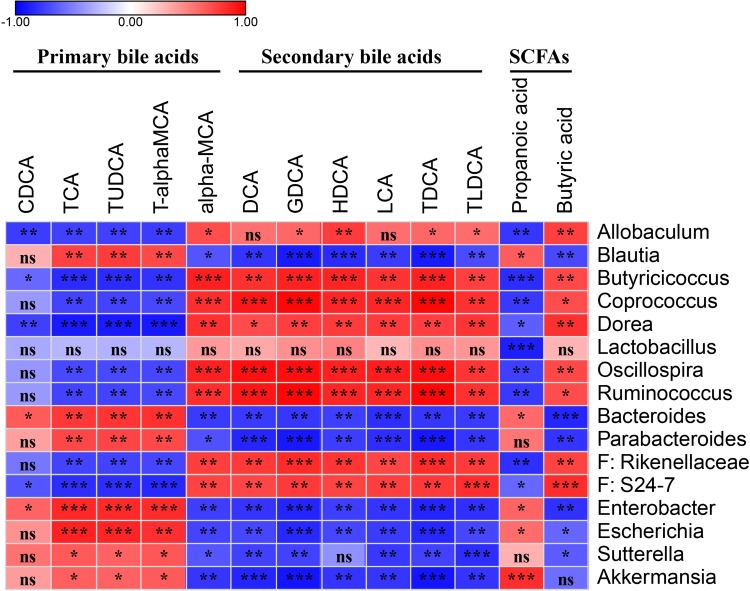
Spearman’s correlation map of the relative abundances of the discrepant OTUs and the levels of the discrepant bile acids and SCFAs among the fecal samples from the PBS and probiotics groups. The *r*-values are denoted with graduated colors, and red and blue grids indicate positive and negative correlations, respectively. “ns” indicates nonsignificance; ^∗^*P* < 0.05, ^∗∗^*P* < 0.01, ^∗∗∗^*P* < 0.001.

To demonstrate whether the key OTUs of the gut microbiota were associated with the discrepant bile acid level, an association analysis was performed based on the Spearman’s rank correlation coefficient. Figure 5 shows primary bile acid TCA, TUDCA, and T-α-MCA were significantly negatively correlated with genera of *Allobaculum*, *Butyricicoccus*, *Coprococcus*, *Dorea*, *Oscillospira*, and *Ruminococcus*, and the families Rikenellaceae and S24-7, and were significantly positively correlated with *Blautia*, *Bacteroides*, *Parabacteroides*, *Enterobacter*, *Escherichia*, *Sutterella*, and *Akkermansia.* Additionally, CDCA was negatively correlated with *Allobaculum*, *Butyricicoccus, Dorea*, and S24-7, and positively correlated with *Bacteroides* and *Enterobacter* (Figure 5 and Supplementary Table S3).

Six secondary bile acids, including DCA, GDCA, HDCA, LCA, TDCA, and TLDCA, and the primary bile acid α-MCA were significantly negatively correlated with *Blautia*, *Bacteroides*, *Parabacteroides*, *Enterobacter*, *Escherichia*, *Sutterella*, and *Akkermansia*, and significantly positively correlated with *Allobaculum*, *Butyricicoccus*, *Coprococcus*, *Dorea*, *Oscillospira*, *Ruminococcus*, Rikenellaceae, and S24-7 (Figure 5 and Supplementary Table S3).

Two discrepant SCFAS demonstrated opposite correlations with the key OTUs. Propanoic acid, which exhibited a similar trend as the primary bile acids, was significantly negatively correlated with *Allobaculum*, *Butyricicoccus*, *Coprococcus*, *Dorea*, *Lactobacillus, Oscillospira*, *Ruminococcus*, Rikenellaceae, and S24-7, and was significantly positively correlated with *Blautia*, *Bacteroides*, *Enterobacter*, *Escherichia*, and *Akkermansia*. Butyric acid demonstrated a similar trend as the secondary bile acids in the correlation analysis (Figure 5 and Supplementary Table S3).

## Discussion

Dysbiosis, defined as “a condition with imbalance in the composition of the bacterial microbiota; this includes an outgrowth of potentially pathogenic bacteria and/or a decrease in bacterial diversity and bacteria beneficial to the host” by Honda K and Littman D R (Honda and Littman, 2012). The dysbiosis of the gut microbiota caused by the misuse of antibiotics is a major factor in the infection of *C. difficile*. The restoration of dysbiosis is an effective measure in the control of CDI. In this study, our probiotics mixture could attenuate *C. difficile* in the feces probably by increasing the relative abundance of Firmicutes and Bacteroidetes and decreasing the abundance of Proteobacteria and Verrucomicrobia. Interestingly, 9 consecutive days of administration of the probiotics mixture, containing five *Lactobacilli* and two *Bifidobacterium* strains, did not increase the abundance of *Lactobacilli* and *Bifidobacterium* (Supplementary Figure S2) in feces and similar result was observed in our previous study (Li et al., 2019). Intake of probiotics mixture may have an effect on gut microbiota and bile acids by their metabolites as transient stay of them in intestine, which would be confirmed in the future study. Persistent supplement of probiotics is one of the shortcomings because the probiotics can’t inhabit the intestine for a long period. Novel probiotic with core bacteria composition of feces besides *Lactobacilli* and *Bifidobacterium* strains is deserve to explore for better colonization of probiotic and restoration of dysbiosis.

Our results showed *Butyricicoccus*, *Coprococcus*, *Dorea*, *Oscillospira*, and *Ruminococcus* were significantly negatively correlated with *C. difficile* numbers, while *Akkermansia*, *Bacteroides*, *Enterobacter*, and *Escherichia* were significantly positively correlation. These negatively correlated bacteria may play a role in the reduction of *C. difficile* numbers in the feces caused by probiotic treatment. Consistent with our results, *Lactobacillus fermentum* could alter the gut microbiota structure by increasing the relative abundance of *Oscillospira* (Lye et al., 2017) and oral probiotic cocktail of *Lactobacillus* and *Bifidobacterium* increased the level of *Ruminococcus* in feces after 8 weeks (De Wolfe et al., 2018). Besides, *Coprococcus* and *Dorea* were also reported to be lower level of abundance in *C. difficile*-infected patient (Shankar et al., 2014; Sokol et al., 2018). In addition, the positively correlated genera *Enterobacter* and *Escherichia* were also found to have a higher relative abundance in CDAD subjects compared to healthy controls (Ling et al., 2014). However, some studies are inconsistent with our results. *Butyricicoccus* was enriched in children with CDAD (Ling et al., 2014) and the abundance of *Akkermansia* in patients with CDI was decreased (Rodriguez et al., 2016). Shahinas reported a negative correlation between CDI and *Bacteroides* (Shahinas et al., 2012). These opposing results may be attributed to the differences in the mouse models, *C. difficile* strain, and patients.

A recent study showed that bile acids played crucial roles in the treatment of CDI by FMT (Mullish et al., 2019). Previous studies showed that bile acids affected the germination and growth of *C. difficile*. TCA, as one of the most highly concentrated primary bile acids in mice (Ma et al., 2018), that is typically used as an important component in *C. difficile* growth media, can promote spore germination in *C. difficile* (Sorg and Sonenshein, 2008). DCA, a secondary bile acid, is toxic to vegetative *C. difficile*. Another secondary bile acid LCA is an inhibitor of spore germination (Britton and Young, 2014). Generally, primary bile acids can promote the growth of *C. difficile*, while secondary bile acids inhibit the growth of *C. difficile*, which have been proved both *in vitro* and *in vivo* (Sorg and Sonenshein, 2010; Buffie et al., 2015). Our data showed that the primary bile acids CDCA, TCA, TUDCA, and T-α-MCA were significantly decreased, while the secondary bile acids DCA, GDCA, HDCA, LCA, TDCA, and TLCA were markedly increased in the mouse feces after treatment with probiotics. Interestingly, α-MCA, one of the primary bile acids in mice, was markedly increased compared to PBS-treated mice. The reason why the result of α-MCA was opposite to the other four primary bile acids is unknown. It was also reported that muricholic acids, including α-MCA, inhibited *C. difficile* spore germination and growth (Francis et al., 2013). Therefore, the change in bile acid profiles characterized as higher level of secondary bile acid and lower level of primary bile acid might contribute to the decrease of *C. difficile* although the other mechanism may also be involved. Spearman’s rank correlation coefficients also confirmed the correlation of *C. difficile* number and bile acids.

*Lactobacillus* species, *Bifidobacterium* species, and other probiotics can ameliorate disease severities in colitis models by increasing the expression level of anti-inflammatory cytokines (Hart et al., 2004) and decreasing the production of inflammatory cytokines (Owaga et al., 2015). Our results showed that there were no significant differences in the expression of two intestinal mucous proteins and most inflammatory related genes between PBS and probiotic group, except that significantly lower levels of IL-12 and reg3β were observed in probiotics-treated group. Some probiotics have the property of decreasing inflammatory factors. *Lactobacillus plantarum* CAU1055 had significantly reduced levels of TNF-α, and IL-6 in a dextran sulfate sodium-induced colitis animal model (Choi et al., 2019). *Bifidobacterium animalis* MB5 and *Lactobacillus rhamnosus* GG prevented the *Escherichia coli*-induced increased expression of IL-1β and TNF-α in Caco-2 cells (Roselli et al., 2006). The probiotics mixture was unable to inhabit the intestine for a long period, and has little effect on the intestinal mucosa, which may be the reason that intestinal mucous proteins, *muc2* and *muc3* had little changed.

However, our conclusion of 7-probiotics consortium decreasing the *C. difficile* based on the mouse model does not accurately mimic *C. difficile* colonization in humans. It is unknown whether the 7-probiotics consortium is effective in patients with CDI. Additionally, two strains of five *Lactobacilli* derived from Tibet wild marmots, further safety evaluation need to be conducted in the future.

## Data Availability Statement

The datasets generated for this article can be found in the NCBI SRA database, accession number: SRP200838.

## Ethics Statement

The studies involving human participants were reviewed and approved by the Ethics Review Committee of the National Institute for Communicable Disease Control and Prevention at the Chinese Center for Disease Control and Prevention. Written informed consent to participate in this study was provided by the participants’ legal guardian/next of kin. The animal study was reviewed and approved by the Ethics Review Committee of the National Institute for Communicable Disease Control and Prevention at the Chinese Center for Disease Control and Prevention.

## Author Contributions

XL, QC, and ZR conceived and designed the experiments. QC, YX, SZ, LS, YH, YK, and SL performed the experiments. XL and YH analyzed the data. XL, QC, JX, and ZR discussed the results. XL and ZR wrote the manuscript.

## Conflict of Interest

The authors declare that the research was conducted in the absence of any commercial or financial relationships that could be construed as a potential conflict of interest.

## Footnotes

1 http://huttenhower.sph.harvard.edu/galaxy/

## Supplementary Material

FIGURE S1 LEfSe taxonomic cladogram generated from 16S rRNA gene sequences and the relative abundance of the phyla. In cladogram **(A)**, the red color indicates the enriched taxa in the control group; green indicates the enriched taxa in the PBS group; and blue indicates the enriched taxa in the probiotics group. The relative abundance of Firmicutes **(B)**, Bacteroidetes **(C)**, Proteobacteria **(D)**, and Verrucomicrobia **(E)** obtained from the LEfSe results.

FIGURE S2 The copies of *Lactobacillus* spp. and *Bifidobacterium* spp. per 200 mg feces as determined by RTq-PCR. **(A,B)** represent *Lactobacillus* and *Bifidobacterium* genus, respectively. The data were performed using one-way ANOVA (*P* < 0.05). All data are expressed as mean ± SD (*n* = 6). ^∗∗∗^*P* < 0.001.

TABLE S1 The names and sequences of primers and probes.

TABLE S2 The concentration of bile acids of PBS and probiotics groups (ng/mg).

TABLE S3 Spearman’s correlation between key OTUs and bile acids and SCFAs.

## Abbreviations

CA: cholic acid
CDCA: chenodeoxycholic acid
GCA: glycocholic acid
GCDCA: glycochenodeoxycholic acid
TCA: taurocholate acid
TUDCA: tauroursodeoxycholic acid
T α-MCA: tauro- α-muricholic acid
α-MCA: α muricholic acid
beta-MCA: beta muricholic acid
DCA: deoxycholic acid
DHCA: dehydrocholic acid
GDCA: glycodeoxycholic acid
GUDCA: glycoursodeoxycholic acid
HDCA: hyodeoxycholic acid
LCA: lithocholic acid
TDCA: taurodeoxycholic acid
THDCA: taurohyodeoxycholic acid
TLCA: taurolicholic acid
UDCA: ursodeoxycholic acid.
