# Supplementary figures and images for: Consortium of Probiotics Attenuates Colonization of Clostridioides difficile
Source: Front Microbiol. 2019 Dec 12;10:2871. doi: 10.3389/fmicb.2019.02871 (PMC6920126; doi:10.3389/fmicb.2019.02871)

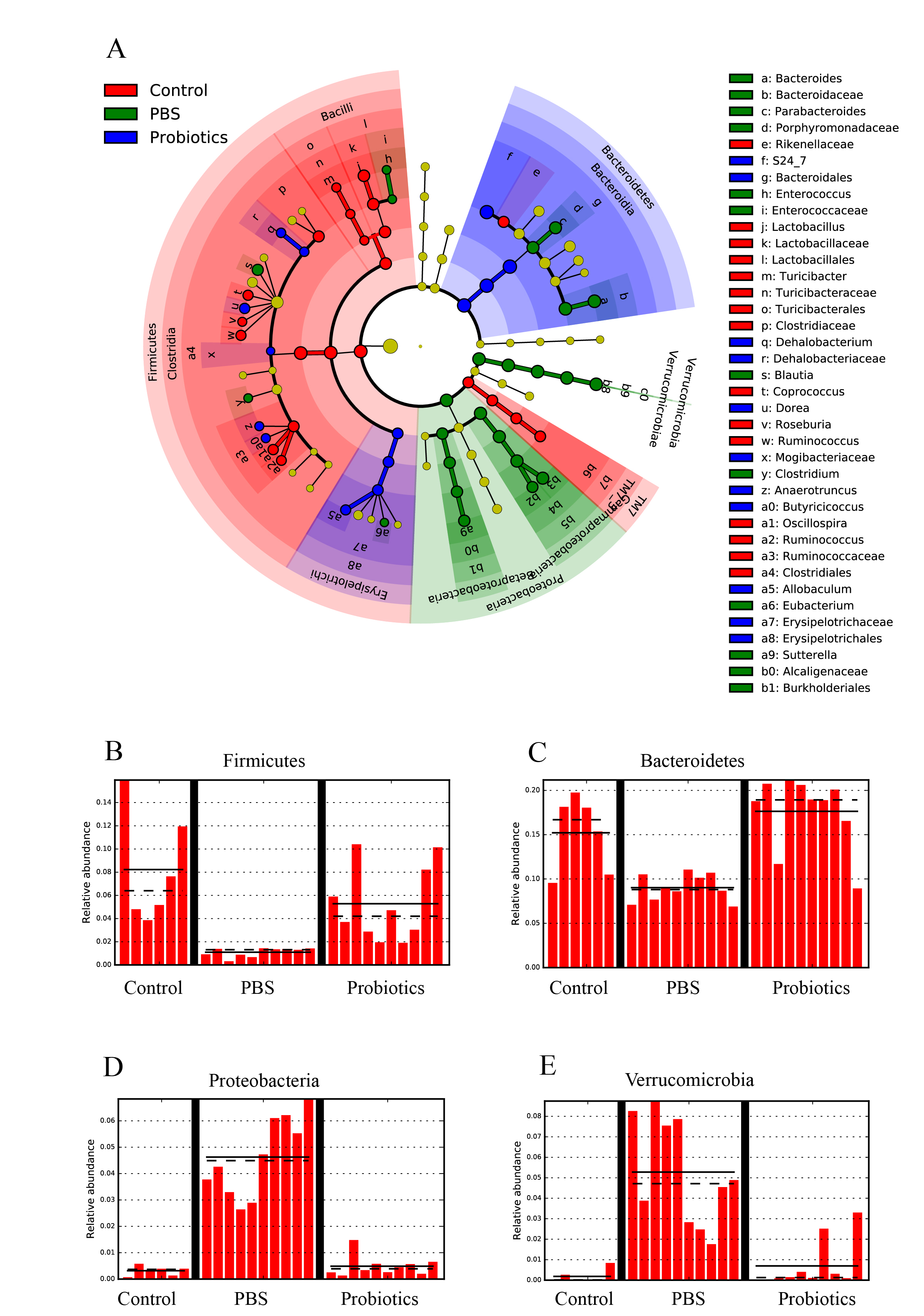

Supplement: FIGURE S1 — LEfSe taxonomic cladogram generated from 16S rRNA gene sequences and the relative abundance of the phyla. In cladogram (A), the red color indicates the enriched taxa in the control group; green indicates the enriched taxa in the PBS group; and blue indicates the enriched taxa in the probiotics group. The relative abundance of Firmicutes (B), Bacteroidetes (C), Proteobacteria (D), and Verrucomicrobia (E) obtained from the LEfSe results. [file Image_1.JPEG]

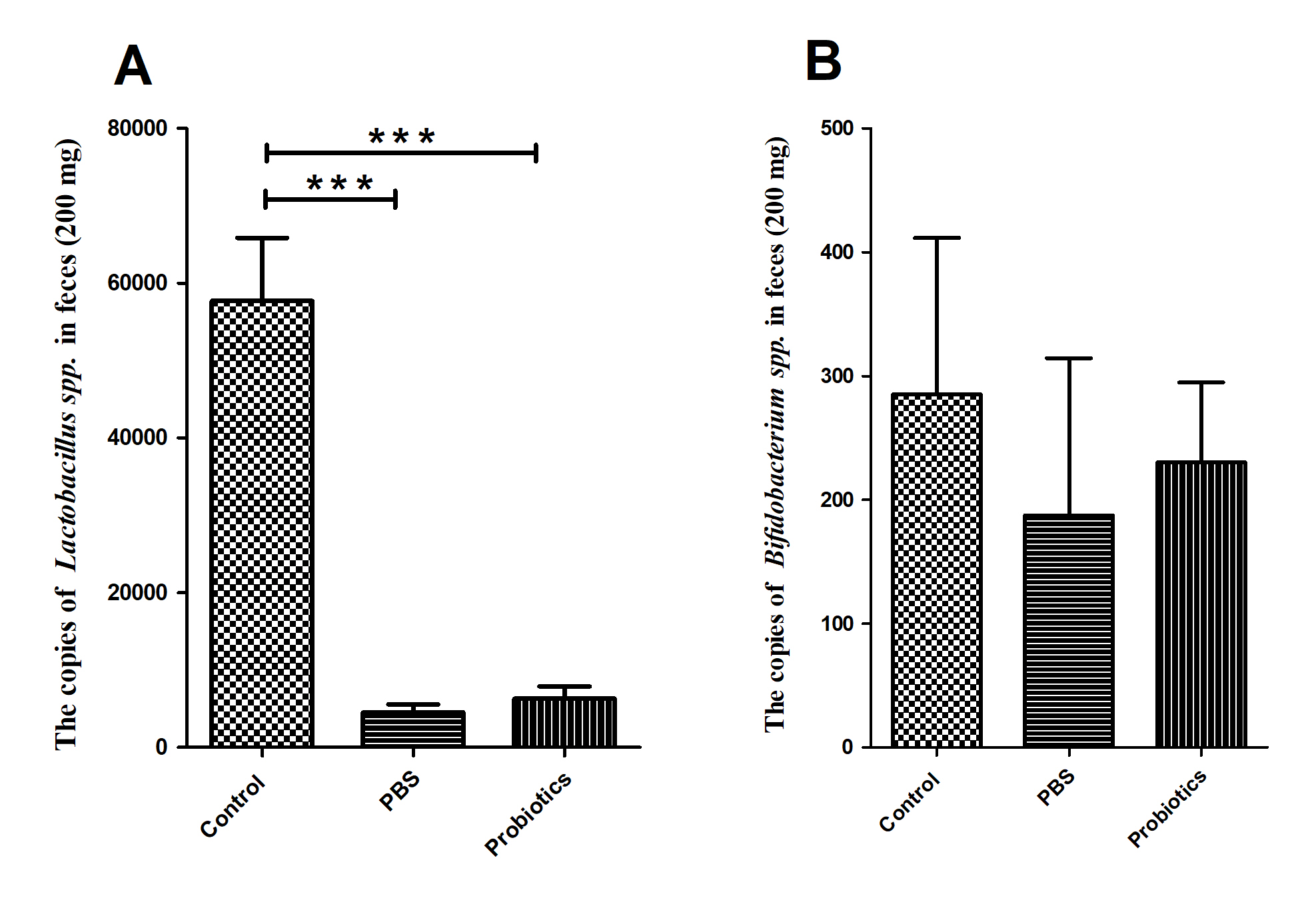

Supplement: FIGURE S2 — The copies of Lactobacillus spp. and Bifidobacterium spp. per 200 mg feces as determined by RTq-PCR. (A,B) represent Lactobacillus and Bifidobacterium genus, respectively. The data were performed using one-way ANOVA (P < 0.05). All data are expressed as mean ± SD (n = 6). ∗∗∗P < 0.001. [file Image_2.JPEG]
